# Dementia Automobilis

**Published:** 1914-05

**Authors:** 


### Dementia Automobilis.

All new editions of our medical dictionaries contain a number of new words and new terms, necessarily essential by reason of the progressive and changing nature of the science and art of medicine, and the term at the head of this article is suggested as one that is greatly needed at the present time, in order to complete and perfect the etiological factors of death in our vital statistics and mortality lists.

The exhilarating effects—amounting in many cases to a veritable intoxication—of high speed and rapid motion that can be attained by the automobile is daily adding to the death rate in a most alarming manner. For both pleasure and business "the horseless carriage" may well be regarded as one of the great advances of the age, and especially is it a great boon to the medical profession; not only for its convenience and comfort, but even by a reasonable degree of speed, greatly extending the field and increasing the capabilities and possibilities of the practioner of medicine and surgery.

And yet, how often it is that one of the genus homo—male or female—at other times and on other occasions reasonably rational, on grasping the steering wheel of an auto and taking charge of the machine, loses control of himself or herself; lose their heads and are positively crazy, endangering not only their lives and those riding with them, but the lives of others who have an equal right to use the public highway.

This speed mania, or may we not term it *"dementia automobilis,"* attacks all classes, men, women and children—the white or black chauffeur, the merchant, the manufacturer, the artisan, the mechanic, the judge and juror, the banker, broker, butcher, baker, tailor, plumber, or tinker—even a Methodist preacher on his way to prayer meeting, going at a thirty or forty-mile clip, not many months ago ran into the writer, greatly endangering four lives lives—the autoist and his companion and the writer and his daughter escaping very serious injury by the veriest miracle; and not very long since was the report in the daily press of the demolition of a $4,500 car, containing the wife and children of a physician, driven by him at 50 miles an hour, rounding a curve, resulting in the death of three, the permanently crippling of two, and when the wreckage was cleared away it was found that one of the steel springs of the car had been driven to the depth of eigth inches into the trunk of a tree by the wayside. And what, if you or I, your wife, son, or daughter, or any one else had been standing there!

Zeppelin airships have carried over 10,000 persons without injuring one of them, not because air-sailing in Germany is less risky than motoring in this country, but because only sane and sound persons are allowed control of the steering gear of an airship; while children, drunkards, fools, and the (temporarily) insane are permitted to drive automobiles. Thirty, forty, and sixty or more miles per hour should never be permitted, unless running on a steel track, with flanged wheels and an absolute "right of way." The pneumatic tire, which may explode or rupture while running at high speed over a very small elevation or depression; the peculiar steering gear with its double-pointed axle, and the rights of others to the public thoroughfares, imperatively demand a more rigid regulation of the speed of automobiles than has heretofore obtained. The high speed of which the auto is capable tempts the too self-reliant driver to indulge in a rapidity of motion and momentum for which no city street or rural roadway is suitable, and which makes homicide too imminent and frequent, occasioning the diurnal report in the columns of the secular press of accidents, injuries, death, and destruction of and by automobiles.

Oh, yes! The auto is a splendid machine, its value is incalculable, its benefits immeasurable, it is being improved every day, and we can no more get along without it than we can do without the telegraph, the telephone, the sewing machine, the Mergenthaler, and the thousands and thousands of latter-day inventions and improvements; but for Heaven's sake, and for the good of mankind, and especially women and children, for the sake of the lame, the deaf and the blind, let us be reasonable in its use; and let the strong arm of the law prevent its abuse.—*Southern Practioner.*

